# From Insect Bites to a Skin Autoimmune Disease: A Conceivable Pathway to Endemic Pemphigus Foliaceus

**DOI:** 10.3389/fimmu.2022.907424

**Published:** 2022-05-27

**Authors:** Ning Li, Valeria Aoki, Zhi Liu, Phillip Prisayanh, Jesus G. Valenzuela, Luis A. Diaz

**Affiliations:** ^1^Department of Dermatology, University of North Carolina at Chapel Hill, Chapel Hill, NC, United States; ^2^Department of Dermatology, Faculdade de Medicina Facultade de Medicina, Universidade de Sao Paulo (FMUSP), Universidade de Sao Paulo, Sao Paulo, Brazil; ^3^Vector Molecular Biology Section, Laboratory of Malaria and Vector Research, National Institute of Allergy and Infectious Diseases, National Institutes of Health, Rockville, MD, United States

**Keywords:** fogo selvagem (FS), Tunisian pemphigus foliaceus, autoantibodies, desmoglein 1, sandflies

## Abstract

In the endemic variants of pemphigus foliaceus (PF), in Brazil and Tunisia, patients generate pathogenic IgG4 anti-desmoglein 1 autoantibodies. Additionally, these patients possess antibodies against salivary proteins from sand flies that react with Dsg1, which may lead to skin disease in susceptible individuals living in endemic areas. This minireview focuses on recent studies highlighting the possible role of salivary proteins from *Lutzomyia longipalpis* (*L. longipalpis*) in EPF from Brazil and *Phlebotomus papatasi* (*P. papatasi*) in EPF from Tunisia. We will briefly discuss the potential mechanisms of molecular mimicry and epitope spreading in the initiation and development of endemic PF (EPF) in Brazil and Tunisia.

## Introduction

Autoimmune diseases affect more than 2.5% of populations (1). Both genetic and environmental factors drive the development of autoimmune diseases. Loss of immunological tolerance to self-antigens results in autoimmunity. Autoantibodies could have evolved during B cell clonal expansion, activated either by self-antigens or by foreign antigens. Molecular mimicry (shared immunologic epitope between foreign and self-antigens) is a general mechanism to explain how exogenous antigens can trigger a specific autoimmune response (2, 3). Infections could induce autoimmune diseases by molecular mimicry between microbes and self-antigens. For instance, molecular mimicry between microbes and certain self-antigens may be implicated in the development of rheumatic fever (4) and rheumatoid arthritis (5, 6). Epitope spreading (ES) may be another fundamental mechanism of the immune responses to foreign antigens relevant in autoimmunity (7–10). ES is utilized by the immune system to diversify the B cell and T cell responses to specific regions of antigens and, in some cases, to cross-reactive epitopes located on unrelated proteins (mimicry). From this initial response, the recognition units of the immune system spread their reactivity to epitopes within the initial antigen (intramolecular) or to epitopes located in neighboring molecules (intermolecular). In some cases, ES involves previous sensitization of T cells by cryptic neoantigens released of apoptosis or inflammation of local tissues or organs. Additionally, T cells may be activated not by antigenic epitopes but rather by local inflammatory cytokines. The term “bystander activation” has been used for this process (9, 10). The possible mechanisms of ES in autoantibody-mediated blistering diseases of the skin have been thoroughly reviewed previously (11, 12).

The environmental inducers of most autoimmune diseases remain to be fully disclosed (13); hence, identifying these triggers is important for closing a knowledge gap and developing strategies to avoid or eliminate the triggers for disease prevention and for novel therapeutic approaches.

## The Endemic Variants of Autoimmune Pemphigus Foliaceus

### The Endemic PF From Brazil

The endemic form of PF, also known as Fogo Selvagem (FS), has been reported in certain subtropical regions of Brazil since the beginning of the 20^th^ century (14). FS shows several unique epidemiologic features, such as the geographic and temporal clustering of patients, an increased frequency of familial patients among young adults and children (15), and an association with certain distinct HLA-DR alleles DRB1*0404, 1402, or 1406 (16). Like the nonendemic forms of PF seen in the USA and worldwide, FS is characterized by subcorneal blisters with acantholysis and pathogenic IgG4 autoantibodies that recognize conformational and calcium-dependent epitopes on the ectodomain of desmoglein 1 (Dsg1), a key desmosomal adhesion molecule in the epidermis (15, 17–24). Purified IgG4 and its F(ab’)2 and Fab’ fragments from FS anti-Dsg1 IgG fractions were pathogenic using the passive transferred mouse model (25, 26). T cells from FS patients also recognize Dsg1 and produce IL-4, IL-5, and IL-6, but not γ-IFN, which this response has a Th2-like cytokine profile (27).

Desmosomal cadherins, i.e., desmogleins (Dgs) Dsg1, Dsg2, Dsg3, and Dsg4 and desmocollins (Dsc) Dsc1, Dsc2, and Dsc3 are critical for epidermal integrity (28, 29). The ectodomain of cadherins is composed of five domains (EC1-EC5), and the C-terminal EC5 is proximal to the membrane with no significant homology to the four “cadherin repeats” (EC1-EC4). Harrison et al. have convincingly demonstrated that the interaction between Dsg and Dsc is heterophilic, with their ectodomains intermingling with each other by *trans* adhesive forces that generate epidermal cell-cell adhesion (29). It is thought that tryptophan 2 (Trp 2) from one desmosomal cadherin monomer of a cell insert into an “acceptor pocket” made, in part, by the RAL (Dsg) or YAT (Dsc) hydrophobic residues from an opposite monomer by a strand-swap process (30, 31). Mutations of Trp2 or RAL pocket residues impair the adhesive function of these molecules (32–36). Importantly, we determined that pathogenic IgG4 from FS patients binds a conformational epitope on the EC1 domain of Dsg1 that overlaps the “acceptor pocket” or adhesive site of this molecule, thus impairing the heterophilic interaction of Dsg1 and Dsc1 (24). These findings have been confirmed and extended using IgG autoantibodies from patients with active PF, Pemphigus Vulgaris (PV), and pathogenic monoclonal antibodies against the *trans* adhesive site of Dsg1 and Dsg3 (37). The authors concluded that autoantibody-induced steric hindrance, rather than intracellular activation, maybe a relevant mechanism of acantholysis in these patients. Additional mechanisms may operate in the acantholysis induced by PV autoantibodies as reported by Di Zenzo et al. (38). They generated monoclonal antibodies from two PV patients and found that some of these antibodies are pathogenic *in vitro* and *in vivo* and recognize epitopes located on the *cis*-adhesive site of EC1 and EC2 of Dsg3. Hence, it is likely that in PV, the population of pathogenic anti-Dsg3 autoantibodies may include subpopulations that impair the *trans* and *cis* interactions of Dsg3. Original studies by Waschke et al., utilizing atomic force microscopy and IgG from PV and PF patients, showed that PV autoantibodies blocked Dsg3 homophilic *trans-*interactions, whereas PF autoantibodies did not inhibit Dsg1 homophilic interactions (39–41). In conclusion, the autoantibody tools provided by FS, PF, and PV patients are enabling investigators to test the role of desmosomal cadherins in acantholysis, the hallmark of these autoimmune skin diseases.

### The Unique Humoral Autoimmune Response to Dsg1 in FS and Healthy Inhabitants From Endemic Regions of FS

We have focused our studies on the Amerindian reservation of Limao Verde (LV) located in the State of Mato Grosso do Sul, Brazil, where a high prevalence (3.4% population) of FS was reported (19). Remarkably, a significant number (55%) of healthy individuals from LV possess nonpathogenic IgG anti-Dsg1 antibodies (42) . FS possess predominantly IgG4 pathogenic anti-Dsg1 autoantibodies and a few mixed with non-pathogenic IgG1-IgG3 as well (21) (43–46). Moreover, a highly sensitive and specific “IgG4 predictor” of FS was developed by serological analysis of the IgG isotypes of the anti-Dsg1 response from 214 FS patients and 261 normal individuals from endemic areas (46). Using this predictor, we found that approximately 50% of FS patients possess IgG4 anti-Dsg1 autoantibodies from one to seven years before disease onset, during the preclinical stage of the disease (42, 46, 47). We further showed that IgG autoantibodies from active FS patients recognize the EC1 and EC2 domains of Dsg1, whereas antibodies from FS patients prior to disease onset and healthy individuals from LV all recognize the EC5 domain of the molecule (47).

### Anti-Dsg1 Autoantibodies Cross-React With Sand Fly (*Lutzomyia longipalpis*) Salivary Proteins LJM11 and LJM17

Epidemiological studies suggest that hematophagous insect bites are risk factors of FS (48, 49) in FS endemic areas, including sand flies (*L. longipalpis*), kissing bugs (reduviid), and black flies (simuliid). They are vectors of disease-causing parasites in leishmaniasis, Chagas disease, and onchocerciasis, respectively (49–51). We found that sera of these patients possess nonpathogenic IgG antibodies against the EC5 domain of Dsg1 (52), suggesting that insect-derived antigens cross-react with the EC5 domain of Dsg1. Because chronic exposure to environmental allergens or insect saliva usually induce IgE and IgG4 antibody responses (53, 54), we tested the sera of FS and healthy inhabitants from the endemic regions of FS for IgE and IgG4 anti-Dsg1 antibodies. We detected high levels of IgE and IgG4 anti-Dsg1 in both groups that were positively correlated (55, 56).

Since nonpathogenic anti-Dsg1 antibodies were detected in sera of leishmaniasis patients (52), we reasoned that while taking their blood meal in humans, *L. longipalpis* inoculates salivary proteins that induce cross-reactive anti-Dsg1 antibodies. Two of these salivary proteins, L.JM11 and LJM17, were well-characterized and available (57, 58). It was also known that mice (59) and human volunteers (60), immunized by *L. longipalpis* bites, generate specific antibodies to salivary antigens LJM11 and LJM17, and is believed to be markers of chronic exposure of humans to *L. longipalpis* bites (61). We focused our studies on the Amerindian reservation of LV (19), where FS, leishmaniasis, and *L longipalpis* are endemic. Thus, Qian et al. (62) showed that IgG4 and IgE antibodies from FS sera and anti-Dsg1 monoclonal antibodies reacted with salivary gland extracts from *L. longipalpis.* Additionally, they tested a small number of FS sera (n=10) and two FS anti-Dsg1 monoclonal antibodies against recombinant LJM11, LJM17, and LJL143 from *L longipalpis* and found that they recognize only LJM11. By testing a larger set of sera from FS patients (n=68) and normal individuals (n=100) from LV and nonendemic control populations [from Sao Paulo (n=33), USA (100), and Japan (70)], we found that FS and normal settlers from this endemic focus had significantly higher values of IgG4 anti-LJM17 antibodies than nonendemic controls (P < 0.001) (63). LJM11 was also recognized by IgG4 antibodies from sera of FS and endemic normal settlers. The levels of IgG anti-Dsg1, IgG4 anti-LJM17, and anti-LJM11 antibodies correlated positively in normal settlers and patients with FS (63). Further, affinity-purified anti-Dsg1 IgG autoantibodies from sera of an FS patient and a healthy person living in LV cross-reacted with LJM17 and LJM11 (63). These findings suggest cross-reactivity of the anti-Dsg1 and anti-LJM17/LJM11 antibodies was extended to experimental animals. Interestingly, 6 of 10 mice, immunized with LJM17 produced IgG1 antibodies (human IgG4 homolog), strongly cross-reacted with recombinant human Dsg1, LJM17, and LJM11 (63). Dsg1 inhibited the binding of these murine IgG1 antibodies to Dsg1 in a dose-dependent manner, which suggests cross-reactivity of these antibody systems (63). The immune cross-reactivity between LJM17 and Dsg1 was confirmed by immunization of chickens (n=2) and rabbits (n=2), which do not possess IgG subclasses (63).

Thus, these findings (62, 63) strongly suggest that salivary antigens LJM17 and/or LJM11 from *L. longipalpis* elicit a cross-reactive anti-Dsg1 autoantibody response in FS patients and normal settlers from endemic areas of FS.

### Anti-Dsg1 Monoclonal Antibodies From FS Patients Cross-React With LJM11 and Other Cadherins

Qian et al. (64) were successful in producing monoclonal antibodies from B cells of 7 FS patients and B cells from one individual in the pre-clinical stage of the disease, mostly IgM and IgG, and a few IgG4. Two of these mAbs recognized LJM11 (62). They concluded that the anti-Dsg1 response in FS is antigen-driven. Unfortunately, the amounts of mAbs were scarce, applicable to analytical assays, but hindering *in vivo* or *in vitro* testing for pathogenicity. Later, Qian et al. (65) constructed IgG4 phage display libraries using mRNA isolated from Epstein-Barr virus (EBV)-transformed B cells derived from three FS patients. After panning the libraries with either Dsg1 or LJM11, 14 independent mAbs (single-chain fragment variable or scFv) were isolated. Unexpectedly, all the scFv mAbs cross-reacted with both Dsg1 and LJM11, and none showed monospecific reactivity. Potential cross-reactivity to LJM17 was not tested. The V genes of all the mAbs were extensively mutated, suggestive of antigen selection. However, two revertant mAbs (reverted to the germline sequence) still reacted with both Dsg1 and LJM11, suggesting that either LJM11 or Dsg1 or both could drive the development of the cross-reactive antibodies in FS (65).

A recent study by Peng et al. (66) showed that a set of 13 anti-Dsg1 FS mAbs recognized the ectodomains of the desmosomal cadherins (Dsg2-4, Dsc1-3, and E-cadherins) as well as with LJM11. They tested mAbs IgG1 and IgG4 anti-Dsg1 derived from FS patients and IgG1 anti-Dsg1 from a pre-FS. Sequence alignment of the eight cadherins revealed six relatively conserved segments. Peptides corresponding to the six homologous sequences of Dsg1 were synthesized and tested for their binding to the mAbs. Out of the six peptides, only a linear peptide 3 (Dsg1-3 peptide), located on the EC2 domain of Dsg1, showed reactivity with the mAbs. The authors proposed that the homologous epitope (homologs of Dsg1-3 peptide) on each of the cadherin is likely mimicked by LJM11 structurally. As a result, anti-LJM11 antibodies react with all the cadherins by cross-reactivity. Thus, the identified cross-reactive epitope on Dsg1 could be the primary target of anti-LJM11 antibodies, which lead to the initial autoantibody response in FS (66). A limitation of this study is that only mAbs were analyzed. The immunoreactivity of Dsg1-3 peptide with serum IgG of endemic healthy individuals, pre-FS, and FS patients was not tested. It is surprising that all the tested mAbs had the same cross-reactive specificity to eight cadherins and LJM11, and none showed monospecificity. Of note, the mAbs (hybridoma or scFv) were originated from EBV-transformed PBMC of FS patients (64, 65) and EBV transformation may have influenced the FS B cells. Nevertheless, mAbs cloned using these protocols of hybridoma or phage clones may be biased due to technical limitations and are not likely to represent the circulating antibody repertoire (67).

### The Endemic PF in Tunisia

Endemic PF was initially reported in Tunisia in the early 1990s by Morini et al. (68) and Bastuji-Garin et al. (69) and confirmed by clinical features and laboratory findings (histology and immunofluorescence). The frequency of PF cases was higher in young women from certain rural regions of Tunisia as compared with a large area of France, with few cases in children and men, unlike FS in Brazil. Tunisian EPF was also seen in family clusters like FS (70), but the HLA DRB1*03, a marker of Tunisian EPF patients (71), differed from HLA markers of FS (DRB1*0404, 1402, or 1406) (16). Additionally, there was an overlap of endemic PF and leishmaniasis in some geographic areas of Tunisia (72) and Brazil (19), with distinct vectors of the parasitosis (*P. papatasi* in Tunisia and *L. longipalpis* in Brazil).

### Anti-Dsg1 Autoantibody Response in Healthy Tunisian Individuals and Tunisian PF

Kallel-Sellami et al. (73) studied the anti-Dsg1 autoantibody response in Tunisian EPF patients (n=29) and normal individuals from the region (n=179). They found IgG4 anti-Dsg1 autoantibodies in EPF and 17% of the healthy individuals showed IgG2 anti-Dsg1 autoantibodies (73). The same investigators extended these studies to include a set of Tunisian EPF (n=90), healthy controls, unrelated to EPF patients (n=270), and 203 samples from healthy relatives of EPF patients. They found positive anti-Dsg1 IgG autoantibodies in over 80% of the patients, 7.4% of the unrelated control group, and 15.7% in healthy controls related to EPF patients (74). We reported, however, that 55% of normal individuals, relatives of FS showed positive IgG1 anti-Dsg1 tests (21, 42, 44). Toumi et al. (75) found that anti-Dsg1 IgG from healthy Tunisian individuals bound epitopes of the C-terminal extracellular domains (EC3 to EC5). Epitopes recognized by Tunisian PF patients, however, were more widely distributed throughout the extracellular domains, suggesting IgGs against EC1 and EC2 developed during disease progression by ES.

### Connecting Sand Fly Bites to the Anti-Dsg1 Response in Tunisian EPF

In 2007, Sellami et al. (76) began to test the sera of patients with vector transmitted diseases such as cutaneous leishmaniasis (*P. papatasi*) and hydatidosis for anti-Dsg1/Dsg3 autoantibodies. They found positive results in 21.7% (n=23) of serum samples from patients with leishmaniasis and in 40% (n=35) of samples from hydatidosis patients which were mostly IgG1, IgG2, and IgG3. In serological studies conducted in Brazil, we found that 43% (n=88) of the sera of leishmaniasis cases possessed anti-Dsg1 autoantibodies that recognize the EC5 domain of Dsg1 (52). In additional studies, Zaraa et al. (77) tested the sera of Tunisian EPF (n=31), Tunisian zoonotic leishmaniasis (n=60), healthy controls (n=91), and bullous pemphigoid (n=31). They found that 58% of the EPF sera recognize Dsg1 and 26% a salivary gland extract from *P. papatasi*. They also found that 13% of the sera of zoonotic leishmaniasis bound Dsg1 and 53% the salivary extract. A low percentage of control sera bound these antigens.

In a recent study, these investigators have extended their studies to test the role of the salivary protein, PpSP32, from *P. papatasi* as the trigger of the humoral anti-Dsg1 and anti-Dsg3 responses in Tunisian EPF (78). They did not report endemic cases of PV, which is mediated by anti-Dsg3 antibodies. They showed that PpSP32 interacts with human Dsg1 and Dsg3 *in vitro* and interestingly, mice immunized with PpSP32 produce antibodies that recognize not only this protein but also Dsg1 and Dsg3. Since the reactions with Dsg1 and Dsg3 cannot be inhibited by PpSP32, they concluded that the antibodies produced by mice did not cross-react with Dsg1/Dsg3. The authors did not address if these PpSP32 induced anti-Dsg1 and anti-Dsg3 antibodies are independent systems or bear some degree of cross-reactivity. Additionally, they showed that sera from Tunisian leishmaniasis patients exhibited high titers of anti-PpSP32 (n=56), anti-Dsg1 (n=14), and anti-Dsg3 (n=17) antibodies, which positively correlated. Incubation of these sera with PpSP32 protein did not abolish the reactivity with Dsg1 or Dsg3. Marzouki et al. (78) proposed the intriguing hypothesis that B and T cells from susceptible individuals recognize PpSP32/Dsg1 or PpSP32/Dsg3 complexes and become activated, producing anti-Dsg1 and anti-Dsg3 autoantibodies. This assumes that antigen-presenting B cells are already primed for Dsg1 or Dsg3 and the generation of the immunogenic complex in the patient would be a key step in the immunization process. However, it is known if sand flies are solenophagous insects (79) that pierce the epidermis and reach the upper dermis capillaries to feed and deposit their saliva around these capillaries away from the epidermal desmosomal cadherins (Dsg1 & Dsg3) (80, 81). This feeding mechanism would weaken the molecular complex formation as suggested by Marzouki et al. (78), unless there is a soluble pool of these epidermal cadherins that diffuse down to the dermis, which is unknown to occur.

## Conclusions and Future Perspectives

Patients with EPF from Brazil and Tunisia, normal inhabitants, and patients with leishmaniasis who live in the same endemic areas are constantly bitten by *L. longipalpis* and *P. papatasi*, respectively (82). Thus, they are exposed to salivary antigens from these vectors and surprisingly generate anti-Dsg1 and anti-Dsg3 autoantibodies. Although these research studies are exciting, they are preliminary and have not yet fulfilled the criteria for environmentally associated autoimmune diseases (Koch’s postulates) (83) to attribute an etiologic role to the sand fly salivary antigens for EPF. Work is in progress to develop experimental animals that, upon immunization with the sand fly salivary antigens, may duplicate the human autoantibody-mediated skin disease.

**Figure 1:**
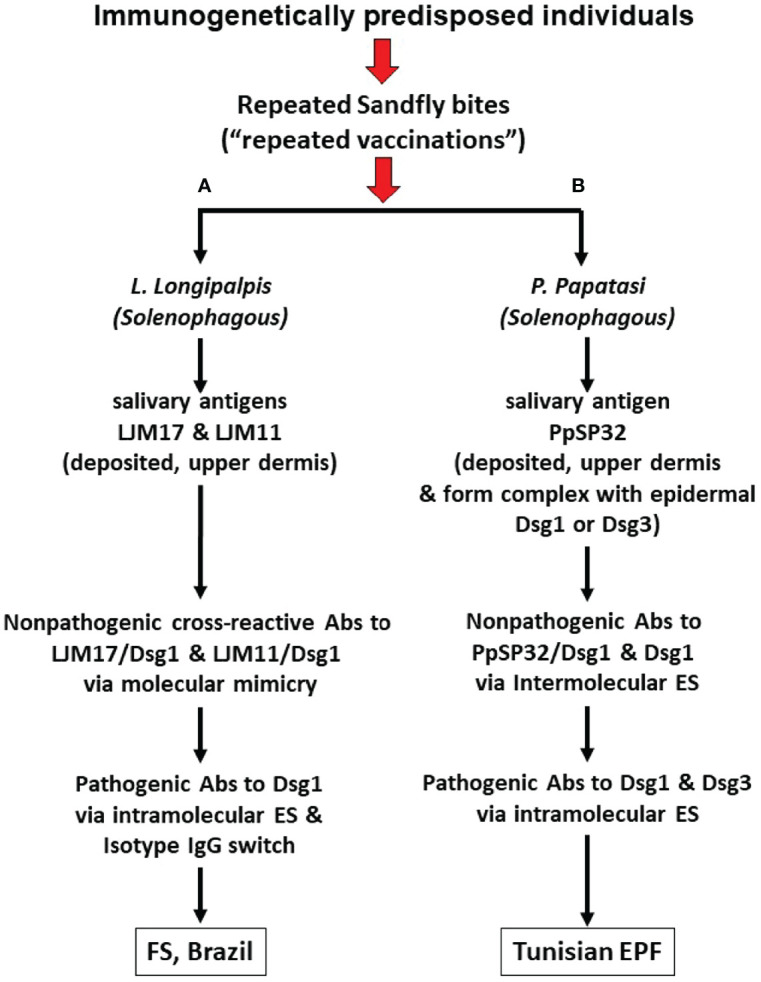
Pathways from insect bites to Endemic Pemphigus Foliaceus (EPF). **(A)** During chronic exposure to sand fly *L. longipalpis* bites, insect salivary proteins including LJM17 and LJM11 are injected into the upper dermis of human skin and elicit a host anti-saliva immune response. Some of the non-pathogenic anti-LJM17 and anti-LJM11 antibodies (Abs) cross-react with human Dsg1 through molecular mimicry. Subsequent intramolecular epitope spreading (ES) and isotype switch, the humoral IgG response will go from non-pathogenic cross-reactive epitopes to pathogenic epitopes of Dsg1 resulting in the production of pathogenic IgG4 autoantibodies (Abs). **(B)** Following exposure to sand fly *P. papatasi* bites, insect salivary proteins including PpSP32 are injected into the upper dermis. PpSP32 protein would interact with Dsg1 and Dsg3 to form immunogenic molecular complexes which elicit an Abs response through the mechanism of intermolecular ES (from the PpSP32 antigen to Dsg1 or Dsg3). These Abs would in time lead to the Tunisian EPF. Experimental data, to date, shows that the cross-reactive Abs against Dsg1 induced by LJM17 and LJM11 from *L. longipalpis* and the Abs against Dsg1 and Dsg3 following immunization with PpSP32 antigen from *P. papatasi* are not pathogenic.

In the FS model (**Figure 1A** and **Table 1**), normal individuals and FS patients sharing the same endemic environment possess autoantibodies against Dsg1 (19–21, 42). In patients, these anti-Dsg1 autoantibodies are IgG4 restricted, pathogenic by passive transfer to neonatal mice, and specific toan epitope of the EC1 domain of Dsg1 (21, 24). During the pre-clinical stage of FS and through clinical remission induced by therapy, the sera possess non-pathogenic IgG1 antibodies against the EC4-5 domain of Dsg1 (47). Importantly, in normal individuals from endemic areas, the non-pathogenic anti-Dsg1 autoantibodies are IgG 1 and bind the EC4-5 epitopes of the antigen (24, 47). The production of pathogenic IgG4 anti-Dsg1 antibodies in FS patients is likely to be the result of the ES mechanism, from an initial reactivity to the EC4-5 of Dsg1 to the EC1 domain where the pathogenic epitopes are located. The ES runs in parallel with the IgG isotype switch from nonpathogenic IgG1 to pathogenic IgG4.

**Table 1:** Sand fly salivary antigens trigger anti-Dsg1 and/or anti-Dsg3 IgG antibody responses.

	Sand fly species, *Solenophagous (* 79, 83)	Salivary antigens	Human seroepidemiologicstudies	Animal immunization studies	Mechanisms ofautoimmunitytriggered by sand flysalivary antigens
**Brazil**	*Lutzomyia longipalpis*	LJM11	The levels of IgG4 anti-LJM11 and anti-Dsg1 were higher and positively correlated in FS and healthy inhabitants of LV (62, 63)	LJM11 induced non-pathogenic IgG anti-Dsg1 response (62, 63)	Cross-reactivity demonstrated by FS IgG mAbs (62, 66)
		LJM17	The levels of IgG4 anti-LJM17 and anti-Dsg1 were higher and positively correlated in FS and healthy inhabitants of LV (63)	LJM17 induced non-pathogenic IgG anti-Dsg1 response (63)	Cross-reactivity demonstrated by affinity-purified serum IgG from immunized mice and inhabitants (normal or FS) of LV (63)
**Tunisia**	*Phlebotomus papatasi* .	PpSP32	The levels of anti- PpSP32, anti-Dsg1, and anti-Dsg3 were higher and positively correlated in blood donors from endemic areas of Tunisian leishmaniasis (78)	PpSP32 induced nonpathogenic IgG anti-Dsg1 and anti-Dsg3 responses (78)	Physical association of foreign and self-antigens (PpSP32/Dsg1 & PpSP32/Dsg3) (78)

Of interest, the sera of patients with leishmaniasis, where *L longipalpis* is the vector, contain non-pathogenic anti-Dsg1 autoantibodies that recognize the EC5 domain of Dsg1 (52). The sera of FS patients and normal individuals, sharing the same endemic environment, also possess IgG4 antibodies against the salivary proteins LJM17 and LJM11 from *L. longipalpis.* These antibodies against LJM17/LJM11 positively correlate with IgG anti-Dsg1 antibodies present in patients and controls (62, 63). Mice, chickens, and rabbits immunized with LJM17/LJM1 produced strong cross-reactive antibodies against Dsg1 (63). The binding of these cross-reactive anti-Dsg1 antibodies to human Dsg1 was inhibited by LJM17, LJM11, and Dsg1 by ELISA. The cross-reactive antibodies, however, were not pathogenic when tested in neonatal mice. Whether the cross-reactive epitopes on the ectodomain of human Dsg1 and *L. longipalpis* LJM17/LJM11 are similar remains to be determined. It is expected that genetically predisposed humans and experimental animals could develop pathogenic anti-Dsg1 autoantibodies if exposed to the appropriate cross-reactive epitopes of Dsg1 and LJM17/LJM11.

In the Tunisian EPF model (**Figure 1B** and **Table 1**), patients and normal individuals sharing the same endemic environment possess autoantibodies against Dsg1 and Dsg3 (78). Interestingly, a significant number of patients with Tunisian EPF and Tunisian leishmaniasis possess anti-Dsg1 IgG autoantibodies and antibodies against the PpSP32 salivary protein from *P. papatasi* that positively correlate. A recent study shows that the PpSP32 associates with Dsg1 and Dsg3 and produces an immunogenic complex that may initiate the autoantibody response in patients and mice. Although the investigators detected anti-Dsg3 antibodies in their Tunisian patients and controls, they did not report endemic cases of PV as reported in Brazil (84). As in Tunisian subjects, anti-Dsg3 autoantibodies were also found in low titers in the Brazilian endemic populations (85, 86), which may be relevant if cases of endemic PV are also identified in Tunisia. Marzouki et al. (78) found no cross-reactivity between antibodies to human Dsg1, or Dsg3, and PpSP32 using inhibition assays adapted to their needs. These results are expected since there is no sequence homology between PpSP32, LJM17/LJM11, and Dsg1. Thus, the anti-Dsg1 and anti-Dsg3 autoantibody response induced by PpSP32 likely resulted from intermolecular ES through the physical association of PpSP32 to epidermal Dsg1 and Dsg3.

In summary, these phenotypes of PF observed in Brazil and Tunisia represent autoimmune diseases where the autoantibody response may be linked to an environmental etiology, i.e., salivary proteins from sand flies autochthonous to these countries. Work is needed to generate an experimental animal that may exhibit the classic clinical and histological features of PF by active immunization with these antigens. These experimental models may offer the opportunity to study the progress of the IgG isotype and IgE responses to these antigens that lead to the generation of pathogenic anti-Dsg1 autoantibodies.

## Author Contributions

All the authors meet all criteria for authorship in the ICMJE recommendations. All authors were involved in the conceptualization, data acquisition, interpretation of data, and writing this mini review. All Authors approved the final submitted version. All the authors agreed to be accountable for all aspects of the work.

## Funding

This research was supported in part by RO1 AR32599 & CTSA-UL1TR002489 (LAD), R01 AI40768 & R01 AI61430 (ZL), CNPq 424536/2018-8 (VA), and the Intramural Research Programs at the National Institute of Allergy and Infectious Diseases, (JGV).

## Conflict of Interest

LAD is co-founder of Epitope Inc. but received no research funding or compensation from this company.

The remaining authors declare that the research was conducted in the absence of any commercial or financial relationships that could be construed as a potential conflict of interest.

## Publisher’s Note

All claims expressed in this article are solely those of the authors and do not necessarily represent those of their affiliated organizations, or those of the publisher, the editors and the reviewers. Any product that may be evaluated in this article, or claim that may be made by its manufacturer, is not guaranteed or endorsed by the publisher.

## Abbreviations

EPF, Endemic pemphigus foliaceus; LV, Limao Verde; Dsg1, Desmoglein 1; Dsc, Desmocollin; L longipalpis, Lutzomyia longipalpis; P papatasi, Phlebotomus papatasi; EC, Extracellular domain; ES, epitope spreading; FS, Fogo Selvagem; mAbs, Monoclonal antibodies.

## References

[1] Ludwig RJ Vanhoorelbeke K Leypoldt F Kaya Z Bieber K McLachlan SM. Mechanisms of Autoantibody-Induced Pathology. Front Immunol (2017) 8:603. doi: 10.3389/fimmu.2017.00603 28620373 PMC5449453

[2] Cusick MF Libbey JE Fujinami RS. Molecular Mimicry as a Mechanism of Autoimmune Disease. Clin Rev Allergy Immunol (2012) 42(1):102–11. doi: 10.1007/s12016-011-8294-7 PMC3266166 22095454

[3] Lee PS Wilson IA. Structural Characterization of Viral Epitopes Recognized by Broadly Cross-Reactive Antibodies. Curr Top Microbiol Immunol (2015) 386:323–41. doi: 10.1007/82_2014_413 PMC4358778 25037260

[4] Cunningham MW. Rheumatic Fever, Autoimmunity, and Molecular Mimicry: The Streptococcal Connection. Int Rev Immunol (2014) 33(4):314–29. doi: 10.3109/08830185.2014.917411 PMC4669348 24892819

[5] Pianta A Arvikar SL Strle K Drouin EE Wang Q Costello CE. Two Rheumatoid Arthritis-Specific Autoantigens Correlate Microbial Immunity With Autoimmune Responses in Joints. J Clin Invest (2017) 127(8):2946–56. doi: 10.1172/JCI93450 PMC5531397 28650341

[6] Rashid T Ebringer A Wilson C. The Link Between Proteus Mirabilis, Environmental Factors and Autoantibodies in Rheumatoid Arthritis. Clin Exp Rheumatol (2017) 35(5):865–71 doi: 10.1007/82_2014_413. 28516867

[7] Mamula MJ. Epitope Spreading: The Role of Self Peptides and Autoantigen Processing by B Lymphocytes. Immunol Rev (1998) 164:231–9. doi: 10.1111/j.1600-065X.1998.tb01223.x 9795779

[8] Vanderlugt CL Miller SD. Epitope Spreading in Immune-Mediated Diseases: Implications for Immunotherapy. Nat Rev Immunol (2002) 2(2):85–95. doi: 10.1038/nri724 11910899

[9] Pacheco Y Acosta-Ampudia Y Monsalve DM Chang C Gershwin ME Anaya JM. Bystander Activation and Autoimmunity. J Autoimmun (2019) 103:102301. doi: 10.1016/j.jaut.2019.06.012 31326230

[10] Brossart P. The Role of Antigen Spreading in the Efficacy of Immunotherapies. Clin Cancer Res (2020) 26(17):4442–7. doi: 10.1158/1078-0432.CCR-20-0305 32357962

[11] Chan LS Vanderlugt CJ Hashimoto T Nishikawa T Zone JJ Black MM. Epitope Spreading: Lessons From Autoimmune Skin Diseases. J Invest Dermatol (1998) 110(2):103–9. doi: 10.1046/j.1523-1747.1998.00107.x 9457902

[12] Didona D Di Zenzo G. Humoral Epitope Spreading in Autoimmune Bullous Diseases. Front Immunol (2018) 9. doi: 10.3389/fimmu.2018.00779 PMC5913575 29719538

[13] Floreani A Leung PSC Gershwin ME. Environmental Basis of Autoimmunity. Clin Rev Allerg Immu (2016) 50(3):287–300. doi: 10.1007/s12016-015-8493-8 25998909

[14] Diaz LA Sampaio SA Rivitti EA Martins CR Cunha PR Lombardi C. Endemic Pemphigus Foliaceus (Fogo Selvagem): II. Current and Historic Epidemiologic Studies. J Invest Dermatol (1989) 92(1):4–12. doi: 10.1111/1523-1747.ep13070394 2642512

[15] Diaz LA Sampaio SA Rivitti EA Martins CR Cunha PR Lombardi C. Endemic Pemphigus Foliaceus (Fogo Selvagem). I. Clinical Features and Immunopathology. J Am Acad Dermatol (1989) 20(4):657–69. doi: 10.1016/S0190-9622(89)70079-7 2654208

[16] Moraes ME Fernandez-Vina M Lazaro A Diaz LA Filho GH Friedman H. An Epitope in the Third Hypervariable Region of the DRB1 Gene is Involved in the Susceptibility to Endemic Pemphigus Foliaceus (Fogo Selvagem) in Three Different Brazilian Populations. Tissue Antigens (1997) 49(1):35–40. doi: 10.1111/j.1399-0039.1997.tb02707.x 9027963

[17] Koulu L Kusumi A Steinberg MS Klaus-Kovtun V Stanley JR. Human Autoantibodies Against a Desmosomal Core Protein in Pemphigus Foliaceus. J Exp Med (1984) 160(5):1509–18. doi: 10.1084/jem.160.5.1509 PMC2187488 6491602

[18] Auad A. Penfigo Foliaceo Sul-Americano No Estado De Goias. Rev Patol Trop (1972) 1:293–346 .

[19] Hans-Filho G dos Santos V Katayama JH Aoki V Rivitti EA Sampaio SA. An Active Focus of High Prevalence of Fogo Selvagem on an Amerindian Reservation in Brazil. Cooperative Group on Fogo Selvagem Research. J Invest Dermatol (1996) 107(1):68–75. doi: 10.1111/1523-1747.ep12298213 8752842

[20] Stanley JR Klaus-Kovtun V Sampaio SA. Antigenic Specificity of Fogo Selvagem Autoantibodies Is Similar to North American Pemphigus Foliaceus and Distinct From Pemphigus Vulgaris Autoantibodies. J Invest Dermatol (1986) 87(2):197–201. doi: 10.1111/1523-1747.ep12695334 3525686

[21] Rock B Martins CR Theofilopoulos AN Balderas RS Anhalt GJ Labib RS. The Pathogenic Effect of IgG4 Autoantibodies in Endemic Pemphigus Foliaceus (Fogo Selvagem). N Engl J Med (1989) 320(22):1463–9. doi: 10.1056/NEJM198906013202206 2654636

[22] Futamura S Martins C Rivitti EA Labib RS Diaz LA Anhalt GJ. Ultrastructural Studies of Acantholysis Induced *In Vivo* by Passive Transfer of IgG From Endemic Pemphigus Foliaceus (Fogo Selvagem). J Invest Dermatol (1989) 93(4):480–5. doi: 10.1111/1523-1747.ep12284041 2778350

[23] Emery DJ Diaz LA Fairley JA Lopez A Taylor AF Giudice GJ. Pemphigus Foliaceus and Pemphigus Vulgaris Autoantibodies React With the Extracellular Domain of Desmoglein-1. J Invest Dermatol (1995) 104(3):323–8. doi: 10.1111/1523-1747.ep12665364 7860995

[24] Evangelista F Roth AJ Prisayanh P Temple BR Li N Qian Y. Pathogenic IgG4 Autoantibodies From Endemic Pemphigus Foliaceus Recognize a Desmoglein-1 Conformational Epitope. J Autoimmun (2018) 89:171–85. doi: 10.1016/j.jaut.2017.12.017 PMC5902409 29307589

[25] Rock B Labib RS Diaz LA. Monovalent Fab' Immunoglobulin Fragments From Endemic Pemphigus Foliaceus Autoantibodies Reproduce the Human Disease in Neonatal Balb/c Mice. J Clin Invest (1990) 85(1):296–9. doi: 10.1172/JCI114426 PMC296418 2295702

[26] Espana A Diaz LA Mascaro JM Jr. Giudice GJ Fairley JA Till GO. Mechanisms of Acantholysis in Pemphigus Foliaceus. Clin Immunol Immunopathol (1997) 85(1):83–9. doi: 10.1006/clin.1997.4407 9325073

[27] Lin MS Fu CL Aoki V Hans-Filho G Rivitti EA Moraes JR. Desmoglein-1-Specific T Lymphocytes From Patients With Endemic Pemphigus Foliaceus (Fogo Selvagem). J Clin Invest (2000) 105(2):207–13. doi: 10.1172/JCI8075 PMC377431 10642599

[28] Delva E Tucker DK Kowalczyk AP. The Desmosome. Cold Spring Harb Perspect Biol (2009) 1(2):a002543. doi: 10.1101/cshperspect.a002543 20066089 PMC2742091

[29] Harrison OJ Brasch J Lasso G Katsamba PS Ahlsen G Honig B. Structural Basis of Adhesive Binding by Desmocollins and Desmogleins. Proc Natl Acad Sci USA (2016) 113(26):7160–5. doi: 10.1073/pnas.1606272113 PMC4932976 27298358

[30] Brasch J Harrison OJ Honig B Shapiro L. Thinking Outside the Cell: How Cadherins Drive Adhesion. Trends Cell Biol (2012) 22(6):299–310. doi: 10.1016/j.tcb.2012.03.004 22555008 PMC3385655

[31] Shapiro L Fannon AM Kwong PD Thompson A Lehmann MS Grubel G. Structural Basis of Cell-Cell Adhesion by Cadherins. Nature (1995) 374(6520):327–37. doi: 10.1038/374327a0 7885471

[32] Chitaev NA Troyanovsky SM. Adhesive But Not Lateral E-Cadherin Complexes Require Calcium and Catenins for Their Formation. J Cell Biol (1998) 142(3):837–46. doi: 10.1083/jcb.142.3.837 PMC2148173 9700170

[33] Kitagawa M Natori M Murase S Hirano S Taketani S Suzuki ST. Mutation Analysis of Cadherin-4 Reveals Amino Acid Residues of EC1 Important for the Structure and Function. Biochem Biophys Res Commun (2000) 271(2):358–63. doi: 10.1006/bbrc.2000.2636 10799302

[34] Pertz O Bozic D Koch AW Fauser C Brancaccio A Engel J. A New Crystal Structure, Ca2+ Dependence and Mutational Analysis Reveal Molecular Details of E-Cadherin Homoassociation. EMBO J (1999) 18(7):1738–47. doi: 10.1093/emboj/18.7.1738 PMC1171260 10202138

[35] Shan WS Tanaka H Phillips GR Arndt K Yoshida M Colman DR. Functional Cis-Heterodimers of N- and R-Cadherins. J Cell Biol (2000) 148(3):579–90. doi: 10.1083/jcb.148.3.579 PMC2174798 10662782

[36] Tamura K Shan WS Hendrickson WA Colman DR Shapiro L. Structure-Function Analysis of Cell Adhesion by Neural (N-) Cadherin. Neuron (1998) 20(6):1153–63. doi: 10.1016/S0896-6273(00)80496-1 9655503

[37] Ishii K Yoshida K Stanley JR Yamagami J Amagai M Ishiko A. Pemphigus Vulgaris and Foliaceus IgG Autoantibodies Directly Block Heterophilic Transinteraction Between Desmoglein and Desmocollin. J Invest Dermatol (2020) 140(10):1919–26.e7. doi: 10.1016/j.jid.2020.02.010 32142800

[38] Di Zenzo G Di Lullo G Corti D Calabresi V Sinistro A Vanzetta F. Pemphigus Autoantibodies Generated Through Somatic Mutations Target the Desmoglein-3 Cis-Interface. J Clin Invest (2012) 122(10):3781–90. doi: 10.1172/JCI64413 PMC3461925 22996451

[39] Waschke J Bruggeman P Baumgartner W Zillikens D Drenckhahn D. Pemphigus Foliaceus IgG Causes Dissociation of Desmoglein 1-Containing Junctions Without Blocking Desmoglein 1 Transinteraction. J Clin Invest (2005) 115(11):3157–65. doi: 10.1172/JCI23475 PMC1242188 16211092

[40] Heupel WM Zillikens D Drenckhahn D Waschke J. Pemphigus Vulgaris IgG Directly Inhibit Desmoglein 3-Mediated Transinteraction. J Immunol (2008) 181(3):1825–34. doi: 10.4049/jimmunol.181.3.1825 18641320

[41] Vielmuth F Spindler V Waschke J. Atomic Force Microscopy Provides New Mechanistic Insights Into the Pathogenesis of Pemphigus. Front Immunol (2018) 9:485. doi: 10.3389/fimmu.2018.00485 29643851 PMC5883869

[42] Warren SJ Lin MS Giudice GJ Hoffmann RG Hans-Filho G Aoki V. The Prevalence of Antibodies Against Desmoglein 1 in Endemic Pemphigus Foliaceus in Brazil. Cooperative Group on Fogo Selvagem Research. N Engl J Med (2000) 343(1):23–30. doi: 10.1056/NEJM200007063430104 10882765

[43] Allen EM Giudice GJ Diaz LA. Subclass Reactivity of Pemphigus Foliaceus Autoantibodies With Recombinant Human Desmoglein. J Invest Dermatol (1993) 100(5):685–91. doi: 10.1111/1523-1747.ep12472348 8491991

[44] Warren SJ Arteaga LA Rivitti EA Aoki V Hans-Filho G Qaqish BF. The Role of Subclass Switching in the Pathogenesis of Endemic Pemphigus Foliaceus. J Invest Dermatol (2003) 120(1):104–8. doi: 10.1046/j.1523-1747.2003.12017.x 12535205

[45] Maldonado M Diaz LA Prisayanh P Yang J Qaqish BF Aoki V. Divergent Specificity Development of IgG1 and IgG4 Autoantibodies in Endemic Pemphigus Foliaceus (Fogo Selvagem). Immunohorizons (2017) 1(6):71–80. doi: 10.4049/immunohorizons.1700029 28868524 PMC5577939

[46] Qaqish BF Prisayanh P Qian Y Andraca E Li N Aoki V. Development of an IgG4-Based Predictor of Endemic Pemphigus Foliaceus (Fogo Selvagem). J Invest Dermatol (2009) 129(1):110–8. doi: 10.1038/jid.2008.189 PMC2736304 18704107

[47] Li N Aoki V Hans-Filho G Rivitti EA Diaz LA. The Role of Intramolecular Epitope Spreading in the Pathogenesis of Endemic Pemphigus Foliaceus (Fogo Selvagem). J Exp Med (2003) 197(11):1501–10. doi: 10.1084/jem.20022031 PMC2193910 12771179

[48] Lombardi C Borges PC Chaul A Sampaio SA Rivitti EA Friedman H. Environmental Risk Factors in Endemic Pemphigus Foliaceus (Fogo Selvagem). "The Cooperative Group on Fogo Selvagem Research". J Invest Dermatol (1992) 98(6):847–50. doi: 10.1111/1523-1747.ep12456932 1593148

[49] Aoki V Millikan RC Rivitti EA Hans-Filho G Eaton DP Warren SJ. Environmental Risk Factors in Endemic Pemphigus Foliaceus (Fogo Selvagem). J Investig Dermatol Symp Proc (2004) 9(1):34–40. doi: 10.1111/j.1087-0024.2004.00833.x 14870983

[50] Brilhante AF Dorval ME Galati EA da Rocha HC Cristaldo G Nunes VL. Phlebotomine Fauna (Diptera: Psychodidae) in an Area of Fishing Tourism in Central-Western Brazil. Rev Inst Med Trop Sao Paulo (2015) 57(3):233–8. doi: 10.1590/S0036-46652015000300009 PMC4544248 26200964

[51] Oliveira AG Galati EA Fernandes CE Dorval ME Brazil RP. Seasonal Variation of Lutzomyia Longipalpis (Lutz & Neiva, 1912) (Diptera: Psychodidae: Phlebotominae) in Endemic Area of Visceral Leishmaniasis, Campo Grande, State of Mato Grosso do Sul, Brazil. Acta Trop (2008) 105(1):55–61. doi: 10.1016/j.actatropica.2007.09.008 18022137

[52] Diaz LA Arteaga LA Hilario-Vargas J Valenzuela JG Li N Warren S. Anti-Desmoglein-1 Antibodies in Onchocerciasis, Leishmaniasis and Chagas Disease Suggest a Possible Etiological Link to Fogo Selvagem. J Invest Dermatol (2004) 123(6):1045–51. doi: 10.1111/j.0022-202X.2004.23438.x 15610512

[53] Manso EC Morato-Castro FF Yee CJ Croce M Palma MS Croce J. Honeybee Venom Specific IgG Subclass Antibodies in Brazilian Beekeepers and in Patients Allergic to Bee Stings. J Investig Allergol Clin Immunol (1998) 8(1):46–51. 9555620

[54] Muller UR. Bee Venom Allergy in Beekeepers and Their Family Members. Curr Opin Allergy Clin Immunol (2005) 5(4):343–7. doi: 10.1097/01.all.0000173783.42906.95 15985817

[55] Qian Y Prisayanh P Andraca E Qaqish BF Aoki V Hans-Filhio G. IgE, IgM, and IgG4 Anti-Desmoglein 1 Autoantibody Profile in Endemic Pemphigus Foliaceus (Fogo Selvagem). J Invest Dermatol (2011) 131(4):985–7. doi: 10.1038/jid.2010.403 PMC3164298 21191415

[56] Qian Y Jeong JS Abdeladhim M Valenzuela JG Aoki V Hans-Filhio G. IgE Anti-LJM11 Sand Fly Salivary Antigen may Herald the Onset of Fogo Selvagem in Endemic Brazilian Regions. J Invest Dermatol (2015) 135(3):913–5. doi: 10.1038/jid.2014.430 PMC4323842 25285921

[57] Valenzuela JG Garfield M Rowton ED Pham VM. Identification of the Most Abundant Secreted Proteins From the Salivary Glands of the Sand Fly Lutzomyia Longipalpis, Vector of Leishmania Chagasi. J Exp Biol (2004) 207(Pt 21):3717–29. doi: 10.1242/jeb.01185 15371479

[58] Xu X Oliveira F Chang BW Collin N Gomes R Teixeira C. Structure and Function of a “Yellow” Protein From Saliva of the Sand Fly Lutzomyia Longipalpis That Confers Protective Immunity Against Leishmania Major Infection. J Biol Chem (2011) 286(37):32383–93. doi: 10.1074/jbc.M111.268904 PMC3173228 21795673

[59] Silva F Gomes R Prates D Miranda JC Andrade B Barral-Netto M. Inflammatory Cell Infiltration and High Antibody Production in BALB/c Mice Caused by Natural Exposure to Lutzomyia Longipalpis Bites. Am J Trop Med Hyg (2005) 72(1):94–8. doi: 10.4269/ajtmh.2005.72.94 15728873

[60] Vinhas V Andrade BB Paes F Bomura A Clarencio J Miranda JC. Human Anti-Saliva Immune Response Following Experimental Exposure to the Visceral Leishmaniasis Vector, Lutzomyia Longipalpis. Eur J Immunol (2007) 37(11):3111–21. doi: 10.1002/eji.200737431 17935072

[61] Teixeira CR Teixeira MJ Gomes RB Santos CS Andrade BB Raffaele-Netto I. Saliva From Lutzomyia Longipalpis Induces CC Chemokine Ligand 2/Monocyte Chemoattractant Protein-1 Expression and Macrophage Recruitment. J Immunol (2005) 175(12):8346–53. doi: 10.4049/jimmunol.175.12.8346 16339576

[62] Qian Y Jeong JS Maldonado M Valenzuela JG Gomes R Teixeira C. Cutting Edge: Brazilian Pemphigus Foliaceus Anti-Desmoglein 1 Autoantibodies Cross-React With Sand Fly Salivary LJM11 Antigen. J Immunol (2012) 189(4):1535–9. doi: 10.4049/jimmunol.1200842 PMC3411885 22798673

[63] Diaz LA Prisayanh P Qaqish B Temple BR Aoki V Hans-Filho G. A Lutzomyia Longipalpis Salivary Protein Induces Cross-Reactive Antibodies to Pemphigus Autoantigen Desmoglein 1. J Invest Dermatol (2020) 140(12):2332–42.e10. doi: 10.1016/j.jid.2020.02.041 32360599

[64] Qian Y Clarke SH Aoki V Hans-Filhio G Rivitti EA Diaz LA. Antigen Selection of Anti-DSG1 Autoantibodies During and Before the Onset of Endemic Pemphigus Foliaceus. J Invest Dermatol (2009) 129(12):2823–34. doi: 10.1038/jid.2009.184 PMC2847628 19571823

[65] Qian Y Jeong JS Ye J Dang B Abdeladhim M Aoki V. Overlapping IgG4 Responses to Self- and Environmental Antigens in Endemic Pemphigus Foliaceus. J Immunol (2016) 196(5):2041–50. doi: 10.4049/jimmunol.1502233 PMC4761459 26826247

[66] Peng B Temple BR Yang J Geng S Culton DA Qian Y. Identification of a Primary Antigenic Target of Epitope Spreading in Endemic Pemphigus Foliaceus. J Autoimmun (2021) 116:102561. doi: 10.1016/j.jaut.2020.102561 33158670 PMC7770069

[67] Chen J Zheng Q Hammers CM Ellebrecht CT Mukherjee EM Tang HY. Proteomic Analysis of Pemphigus Autoantibodies Indicates a Larger, More Diverse, and More Dynamic Repertoire Than Determined by B Cell Genetics. Cell Rep (2017) 18(1):237–47. doi: 10.1016/j.celrep.2016.12.013 PMC5221611 28052253

[68] Morini JP Jomaa B Gorgi Y Saguem MH Nouira R Roujeau JC. Pemphigus Foliaceus in Young Women. An Endemic Focus in the Sousse Area of Tunisia. Arch Dermatol (1993) 129(1):69–73. doi: 10.1001/archderm.1993.01680220081019 8420494

[69] Bastuji-Garin S Souissi R Blum L Turki H Nouira R Jomaa B. Comparative Epidemiology of Pemphigus in Tunisia and France: Unusual Incidence of Pemphigus Foliaceus in Young Tunisian Women. J Invest Dermatol (1995) 104(2):302–5. doi: 10.1111/1523-1747.ep12612836 7829889

[70] Abida O Masmoudi A Rebai A Ben Ayed M Mahfoudh N Kallel-Sellami M. The Familial Feature of Tunisian Endemic Pemphigus Foliaceus. Br J Dermatol (2009) 161(4):951–3. doi: 10.1111/j.1365-2133.2009.09386.x 19673877

[71] Abida O Zitouni M Kallel-Sellami M Mahfoudh N Kammoun A Ben Ayed M. Tunisian Endemic Pemphigus Foliaceus Is Associated With the HLA-DR3 Gene: Anti-Desmoglein 1 Antibody-Positive Healthy Subjects Bear Protective Alleles. Br J Dermatol (2009) 161(3):522–7. doi: 10.1111/j.1365-2133.2009.09207.x 19486004

[72] Ben Rachid MS Ben Ammar R Redissi T Ben Said M Hellal H Bach-Hamba D. [Geography of Major Parasitosis in Tunisia]. Arch Inst Pasteur Tunis (1984) 61(1):17–41. 6535511

[73] Kallel Sellami M Ben Ayed M Mouquet H Drouot L Zitouni M Mokni M. Anti-Desmoglein 1 Antibodies in Tunisian Healthy Subjects: Arguments for the Role of Environmental Factors in the Occurrence of Tunisian Pemphigus Foliaceus. Clin Exp Immunol (2004) 137(1):195–200. doi: 10.1111/j.1365-2249.2004.02493.x 15196262 PMC1809068

[74] Abida O Kallel-Sellami M Joly P Ben Ayed M Zitouni M Masmoudi A. Anti-Desmoglein 1 Antibodies in Healthy Related and Unrelated Subjects and Patients With Pemphigus Foliaceus in Endemic and Non-Endemic Areas From Tunisia. J Eur Acad Dermatol Venereol (2009) 23(9):1073–8. doi: 10.1111/j.1468-3083.2009.03265.x 19453789

[75] Toumi A Saleh MA Yamagami J Abida O Kallel M Masmoudi A. Autoimmune Reactivity Against Precursor Form of Desmoglein 1 in Healthy Tunisians in the Area of Endemic Pemphigus Foliaceus. J Dermatol Sci (2013) 70(1):19–25. doi: 10.1016/j.jdermsci.2013.02.002 23489520 PMC3622174

[76] Sellami MK Zitouni M Tombari W Ben Ayed M Abida O Laadhar L. Anti-Desmoglein-1 Antibodies are Prevalent in Tunisian Patients With Hydatidosis and Leishmaniasis. Brit J Dermatol (2007) 156(3):591–3. doi: 10.1111/j.1365-2133.2006.07687.x 17300263

[77] Zaraa I Boussoffara T Ben Ahmed M Marzouki S Ben Hassouna N Sellami MK. Exposure to Phlebotomus Papatasi and/or Leishmania Major: Possible Etiologic Link to Tunisian Pemphigus. J Invest Dermatol (2012) 132(2):479–82. doi: 10.1038/jid.2011.291 22011908

[78] Marzouki S Zaraa I Abdeladhim M Benabdesselem C Oliveira F Kamhawi S. Implicating Bites From a Leishmaniasis Sand Fly Vector in the Loss of Tolerance in Pemphigus. JCI Insight (2020) 5(23):1–12. doi: 10.1172/jci.insight.123861 PMC7714401 33108348

[79] Lavoipierre MM. Feeding Mechanism of Blood-Sucking Arthropods. Nature (1965) 208(5007):302–3. doi: 10.1038/208302a0 5882467

[80] Ribeiro JMC. Blood-Feeding Arthropods - Live Syringes or Invertebrate Pharmacologists. Infect Agent Dis (1995) 4(3):143–52. 8548192

[81] Ribeiro JM Valenzuela JG Pham VM Kleeman L Barbian KD Favreau AJ. An Insight Into the Sialotranscriptome of Simulium Nigrimanum, A Black Fly Associated With Fogo Selvagem in South America. Am J Trop Med Hyg (2010) 82(6):1060–75. doi: 10.4269/ajtmh.2010.09-0769 PMC2877412 20519601

[82] Aoki V Abdeladhim M Li N Cecilio P Prisayanh P Diaz LA. Some Good and Some Bad: Sand Fly Salivary Proteins in the Control of Leishmaniasis and in Autoimmunity. Front Cell Infect Microbiol (2022) 12:839932. doi: 10.3389/fcimb.2022.839932 35281450 PMC8913536

[83] Miller FW Pollard KM Parks CG Germolec DR Leung PS Selmi C. Criteria for Environmentally Associated Autoimmune Diseases. J Autoimmun (2012) 39(4):253–8. doi: 10.1016/j.jaut.2012.05.001 PMC3468712 22771005

[84] Rocha-Alvarez R Ortega-Loayza AG Friedman H Campbell I Aoki V Rivitti EA. Endemic Pemphigus Vulgaris. Arch Dermatol (2007) 143(7):895–9. doi: 10.1001/archderm.143.7.895 17638734

[85] Arteaga LA Prisayanh PS Warren SJ Liu Z Diaz LA Lin MS. A Subset of Pemphigus Foliaceus Patients Exhibits Pathogenic Autoantibodies Against Both Desmoglein-1 and Desmoglein-3. J Invest Dermatol (2002) 118(5):806–11. doi: 10.1046/j.1523-1747.2002.01743.x 11982757

[86] Hilario-Vargas J Dasher DA Li N Aoki V Hans-Filho G dos Santos V. Prevalence of Anti-Desmoglein-3 Antibodies in Endemic Regions of Fogo Selvagem in Brazil. J Invest Dermatol (2006) 126(9):2044–8. doi: 10.1038/sj.jid.5700388 16763546

